# Short-Term Impact of Dry Needling Treatment for Myofascial Pain on Equine Biomechanics Through Artificial Intelligence-Based Gait Analysis

**DOI:** 10.3390/ani15111517

**Published:** 2025-05-22

**Authors:** María Resano-Zuazu, Jorge U. Carmona, David Argüelles

**Affiliations:** 1 Department of Animal Medicine and Surgery, Faculty of Veterinary Medicine, Universidad de Córdoba, Campus de Rabanales, 14071 Córdoba, Spain; z42rezum@uco.es; 2 Grupo de Investigación Terapia Regenerativa, Departamento de Salud Animal, Universidad de Caldas, Calle 65 No 26-10, Manizales 170004, Colombia; carmona@ucaldas.edu.co

**Keywords:** horse, physiotherapy, trigger points, machine learning, locomotion analysis

## Abstract

Myofascial pain syndrome (MPS) is a common source of musculoskeletal pain, characterized by trigger points (TrPs). In horses, MPS is frequently underdiagnosed, and evidence on DN effectiveness is limited. This study investigated whether DN can improve the biomechanics in horses using an artificial intelligence (AI)-based markerless smartphone application (app). Fourteen horses participated, including nine used in assisted therapy, four leisure horses, and one with mixed use. The presence of TrPs was evaluated in six muscles through manual palpation: brachiocephalicus, trapezius, gluteus medius, biceps femoris, semitendinosus, and quadriceps femoris. The horses were divided into a treatment group (TG) (*n* = 7) and control group (CG) (*n* = 7). Biomechanical data were recorded in a straight line at a trot before the treatment (T0), immediately after the treatment (T1), and 72 h post-treatment (T72). The stride frequency (SF) was significantly lower (*p* < 0.05) at 72 h compared with both before and immediately after the treatment. The SF of the TG at 72 h was significantly lower than the SF of the CG at T1 (*p* < 0.05). Non-significant differences were observed for both the asymmetry push-off and impact phase variables, except for the forelimb head range of motion (FHROM) severity, which was significantly (*p* < 0.05) greater in the CG than in the TG. This study suggests that DN may enhance the gait quality in horses with MPS.

## Simple Summary

The diagnosis and treatment of equine myofascial pain remain challenges in the field of veterinary physiotherapy and rehabilitation. Its high prevalence, underdiagnosis, and the limited scientific literature justify it as a suitable subject for investigation. This study laid a foundation for exploring the effectiveness of one of the most efficient techniques for its treatment. The aim was to determine the effects of dry needling on biomechanical parameters immediately and 72 h after treatment using a mobile application based on artificial intelligence. A significantly lower stride frequency was observed at 72 h compared with both before and immediately after the treatment. The stride frequency of the treatment group at 72 h was lower than that of the control group immediately post-treatment. The forelimb head range of motion severity was significantly greater in the control group than in the treatment group. Integrating dry needling into a multimodal rehabilitation approach, combined with artificial intelligence-based technology, could enhance the management and understanding of myofascial pain in horses.

## 1. Introduction

Musculoskeletal disorders rank among the most prevalent problems in horses, with lameness being the primary clinical sign, often reflecting pain or dysfunction within the musculoskeletal system [1]. Chronic pain-related lameness is often not detected by conventional methods. The most common form of chronic pain is myofascial pain syndrome (MPS) [2]. In veterinary medicine, research on MPS is limited and has primarily focused on dogs and horses [2,3,4,5,6,7,8,9,10,11,12].

MPS is a common cause of musculoskeletal pain, characterized by the presence of trigger points (TrPs), which are hyperirritable spots in a taut muscle band that are painful when compressed, stretched, or contracted, and often cause referred pain at a distance. They can also lead to referred tenderness, motor dysfunction, and autonomic symptoms [13].

Myofascial pain is often overlooked regarding its potential as a cause or contributor to other pain problems, such as cervical and back pain and temporomandibular disorders. TrPs often act as a primary source of dysfunction and can exist without any underlying disease or physical tissue damage [14]. The current diagnostic criteria rely on a detailed history and physical examination [15]. TrPs are commonly identified by feeling via palpation of a taut band and the presence of small areas of muscle known as knots [16]. Both palpation or needle insertion can elicit local twitch responses (LTRs), defined by rapid contractions of muscle fibers, which can lead to symptoms of pain [15]. According to a Delphi study [17], TrPs can be diagnosed if at least two of the following diagnostic criteria are present: a taut band, a hypersensitive spot, and referred pain.

The approach to treating MPS has progressed to include invasive approaches, such as using a needle to treat musculoskeletal pathology in humans [18]. Dry needling (DN) is a therapeutic technique performed with a fine and solid filiform needle for the treatment of TrPs. DN aims to restore body function by rapidly and briefly needling irregular or dysfunctional tissues [19]. The efficacy of DN for MPS has been extensively studied in humans and has become one of the recommended treatments [15,20,21]. To the authors’ knowledge, there are no previously published studies that assessed the effectiveness of the DN technique in horses.

The development and establishment of reliable qualitative and quantitative methods is essential to the evaluation of equine gait patterns [22]. The relationship between competition dressage horse scores and gait characteristics, including stride frequency (SF), has been studied. The results indicate that a reduced SF during walking and trotting correlated with higher scores [23,24].

In humans, clinical gait analysis has been used to guide treatment for over 50 years. Throughout this period, significant advances have been made in motion capture technology and software development, largely due to innovations in biomechanics [25]. The integration of gait analysis with artificial intelligence (AI) has transformed the objectivity and accuracy of gait assessments [26]. The use of AI for patients with gait disorders has been reported [26,27,28,29]. The iPhone application (app) TDPT-GT, a markerless motion capture system based on machine learning, was employed to differentiate between pathological and healthy gait patterns [29].

While the study of biomechanics in horses has captivated scientists for centuries, meaningful advancements only began to materialize in the late twentieth century alongside technological progress [30]. The development of various measurement technologies in this field has been documented [31]. Recent advancements in both hardware and software have facilitated more extensive and sophisticated studies, thereby increasing the accessibility and cost-effectiveness of biomechanical research [30]. Mouloodi et al. [22] reviewed AI and machine learning, which significantly speeds up data analysis and interpretation, and offers significant advantages over traditional statistical tools historically used in equine biomechanical research. An AI-driven program using pose estimation, which has been utilized to analyze human and animal movement without instrumented devices, has shown promising outcomes when applied to horses [32]. An AI-based markerless smartphone app (Sleip, Stockholm, Sweden) was compared with a multi-camera marker-based motion capture system in horses (Qualisys AB, Motion Capture Systems, Göteborg, Sweden). This study concluded that the smartphone measurement tool can effectively detect lameness at relevant levels, proving easy to use for veterinarians [1]. Additionally, another study found that the proportional limits of agreement between the same smartphone app and a validated inertial measurement unit gait analysis system (Xsens MTw) using the MTManager software (v2020.0.2, Xsens, Enschede, The Netherlands), surpassed the current “lameness thresholds” for identifying the affected limb(s) in lame horses [33].

There have been no studies regarding the application of gait analysis to assess the effectiveness of DN in veterinary medicine. In humans, the Qualysis motion analysis system was used to demonstrate the efficacy of DN in human patients with piriformis syndrome. A greater peak hip extension angle during walking was observed after DN treatment compared with the control group [34]. The AccuGait stabilizer platform for assessing postural parameters and the Zebris treadmill for assessing gait parameters were used to measure the effects of DN in patients with chronic low back pain. Significant improvements in functional efficiency and pain reduction were observed [35]. No studies were found that demonstrated the efficacy of DN using AI-based gait analysis in humans or animals.

This study was based on the hypothesis that MPS alters equine gait patterns and that DN improves muscular functionality. Therefore, the aim was to evaluate the effectiveness of the DN technique for treating myofascial pain on equine gait parameters using an AI-based markerless smartphone app.

## 2. Materials and Methods

### 2.1. Horses and Treatment Allocation

In this preliminary clinical study, 14 horses from an equine-assisted therapy center received clinical evaluations during their physiotherapy assessments. The enrolled horses were seven mares and seven geldings of various breeds that were presented for consultation during the study period. They weighed between 378 and 511 kg and ranged in age from 4 to 29 years. Individual data are provided in Supplementary Table S1. Their primary purpose was equine-assisted therapy and recreation. All horses were kept outdoors under similar management conditions. The equine-assisted therapist, responsible for the management of the animals, reported the following main complaints: lack of or irregular hind limb propulsion, toe dragging, excessive weight-bearing in forelimbs, and discomfort in the neck and shoulder area. None of the animals were taking anti-inflammatory or analgesic medications, had received physical therapy in the past week, were pregnant mares, had a history of previously diagnosed lameness, or had neurological disorders. The horses were randomized into two groups of seven: the treatment group (TG), which included horses that received DN treatment (*n* = 7), and the control group (CG), which included horses that received no treatment or sham interventions (*n* = 7).

### 2.2. Muscle Assessment and DN Treatment Technique

The brachiocephalicus, trapezius, gluteus medius, biceps femoris, semitendinosus, and quadriceps femoris muscles on both sides of all horses were evaluated. A detailed description of muscle anatomical references and actions is presented in Appendix A (Table A1).

The brachiocephalicus muscle was assessed using pincer palpation, which entailed grasping the muscle with a pincer to identify TrPs. The other muscles were explored using flat palpation, which involved sliding a fingertip across the muscle fibers of the muscle to find TrPs. When a taut band was identified, it was palpated to locate the most sensitive spot, indicating the presence of a TrP. This finding was corroborated by signs of discomfort in the horse, including alterations in facial expression and movements to avoid contact with the clinician.

The DN informed consent was obtained from the owners. DN was performed with 0.30 mm × 40 mm and 0.25 × 25 mm needles (Agupunt S.L., Barcelona, Spain). The needle sizes used for the treatment of each muscle are presented in Appendix A (Table A1). These needles were specifically designed for DN and manufactured from AISI 304H stainless steel, both in the handle and in the body. They feature a specially sharpened tip design and come equipped with a guide tube.

**Figure 1:**
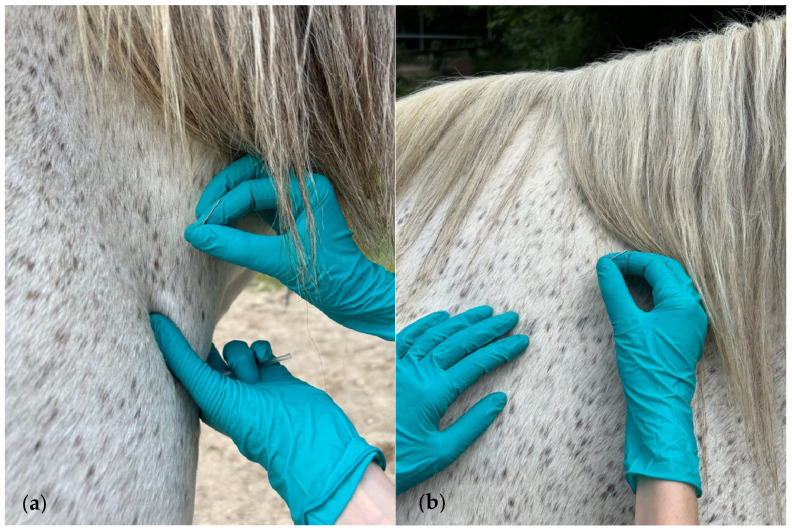
(**a**) Needling the distal part of the brachiocephalicus muscle; (**b**) dry needling (DN) of the cervical part of the trapezius muscle.

The DN treatment was applied to all muscles using the fast-in–fast-out technique described by Hong [36]. According to this technique, the needle is moved in and out in different directions to find sensitive spots in a TrP region in a fast-moving procedure. For the brachiocephalicus muscle, the needle was inserted parallel to the neck in a pincer grip at the distal part of the muscle, directed across its belly toward the taut band (Figure 1a). The cervical part of the trapezius muscle was needled perpendicularly in its dorsal and caudal area toward the supraspinatus fossa (Figure 1b).

**Figure 2:**
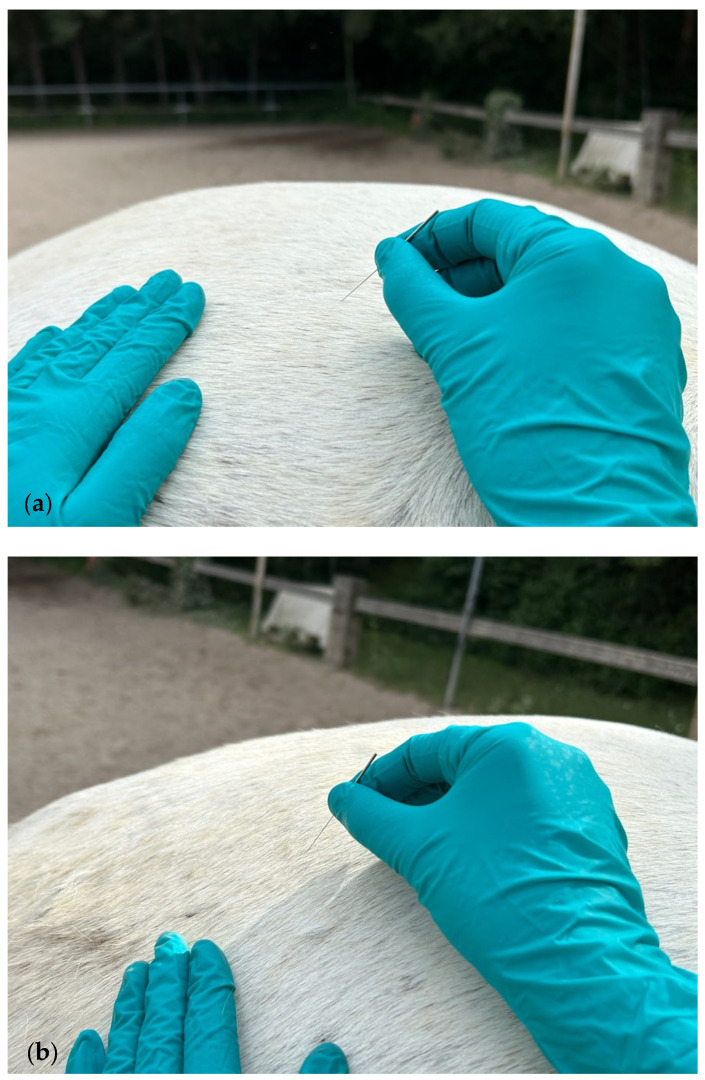
(**a**) DN of the gluteus medius muscle; (**b**) DN of the biceps femoris muscle.

For the gluteus medius and biceps femoris, the needle was inserted perpendicularly to the muscle belly above the coxal tuberosity (Figure 2a,b).

**Figure 3:**
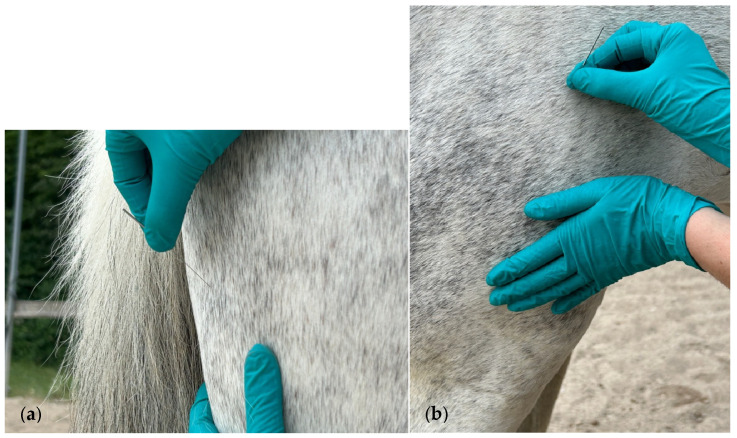
(**a**) Approach of the semitendinosus muscle with DN; (**b**) a safe distance of one hand’s width from the stifle joint before needling the quadriceps femoris muscle.

The semitendinosus was needled perpendicularly to the muscle below the ischial tuberosity (Figure 3a). Lastly, the quadriceps femoris was needled perpendicularly to the muscle belly, through the vastus lateralis head, one hand’s width proximal to the stifle joint (Figure 3b).

### 2.3. Data Collection and Biomechanical Analysis

**Figure 4:**
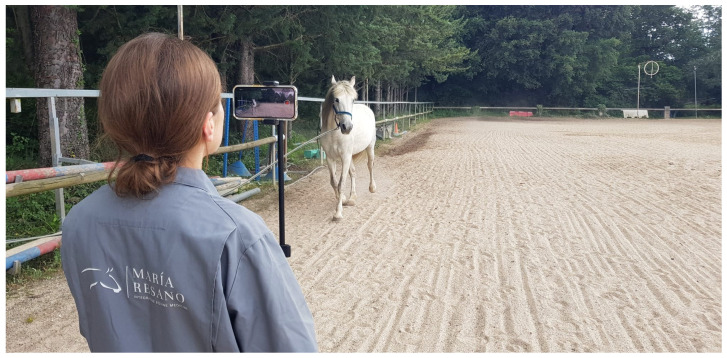
Motion capture during straight-line trot using a markerless smartphone application based on artificial intelligence (AI).

Biomechanical data were collected using an iPhone 14 (v17.5.1 (Apple, Cupertino, CA, USA)) running the Sleip smartphone app (v3.5.0.5, Sleip, Stockholm, Sweden). The smartphone was positioned approximately 1.5 m off the ground on a tripod (model RXPH61, Rixus, IJsselstein, The Netherlands) oriented toward the horse. Recording was performed by rotating the phone to landscape mode at eye level (Figure 4).

All horses were recorded in the same area of the outdoor arena on a flat surface. The horses were filmed under natural light. Each horse was led by an experienced handler, who was a riding instructor and equine-assisted therapist. A head collar with a lead line was used. The horse was trotted back and forth twice on a 30 m straight line starting from the rear. The horses were led by the same handler except for one control horse that was led by its owner. The horses were trotted at a comfortable and steady speed for both the handler and horse, which allowed the horse’s head to move freely.

### 2.4. Procedure and Outcome Variable Measurements

The muscle evaluation and DN treatment were performed by an equine rehabilitation veterinarian (M.R.Z.) with DN training and professional experience in equine rehabilitation. The muscle evaluation and DN treatment, which consisted of a single session, was performed on the right side of each horse and immediately repeated on the left side of the treatment group. Recordings were made before the DN treatment (T0), immediately after the DN treatment (T1), and 72 h after the DN treatment (T72). The same procedure was used in the control group, which did not receive treatment, i.e., no needle insertion. The measured biomechanical variables were grouped into the stride frequency (SF) (number and variation (low (1), medium (2), and high (3)) of the forelimb and hindlimb strides, and stride frequency ((strides/s) in a straight line at trot), asymmetry (forelimb head range of motion (FHROM) and hindlimb pelvic range of motion (HPROM) (%), and FHROM and HPROM severities (none (0), very mild (1), mild (2), moderate (3), and severe (4) (considering both the impact and push-off phases)). Accordingly, the impact values were visualized in the app as the difference between the two minima in the vertical position of the right and left halves of a stride, while the push-off values were visualized in the app as the difference between the two maxima in the vertical position of the right and left halves of a stride.

### 2.5. Statistical Analysis

The data were analyzed using the free statistical software JASP (version 0.18.3, University of Amsterdam, The Netherlands). Generalized linear mixed models (GLMMs) were used for the analysis. Continuous numerical variables from the objective gait analysis were modeled using the Gaussian distribution family with an identity link function. Discrete numerical variables were evaluated using the Poisson distribution family and the square root link function. The fixed effects in the model included the treatment (two levels: treatment group (TG) and control group (CG)), time (three levels: T0, T1, and T72), and their interaction. Each horse was included as a random factor to account for inter-individual variability.

Model validation was performed by examining residual plots to ensure that the assumptions of normality and homoscedasticity were met for continuous variables. For the Poisson model, dispersion was checked to confirm that the data were not overdispersed, and in cases where overdispersion was observed, a negative binomial model was considered. The goodness of fit was assessed using likelihood ratio tests and the Akaike’s information criterion (AIC) to compare the model fit between nested models. When fixed effects or interactions were statistically significant, post hoc comparisons were performed using Tukey’s test. A *p* < 0.05 was considered statistically significant for all analyses.

The statistical power of this preliminary clinical trial was calculated post hoc using GLIMMPSE (https://glimmpse.samplesizeshop.org (accessed on 2 October 2024)), an online power and sample size calculation tool for GLMMs. The overall power for the study variables was greater than 0.8 (β) at a 0.05 significance level (α), indicating sufficient power to detect meaningful effects in the study parameters.

## 3. Results

A total of 606 stride observations for head movement and 365 stride observations of pelvic movement during the strides of the included horses were recorded using the video app.

### 3.1. Stride Frequency

The treatment and time-fixed factors, along with their interactions, did not significantly influence the number and variation of forelimb and hindlimb strides. However, the SF (strides per second) was significantly impacted by the time factor and the interaction between the treatment and time (Table 1).

**Figure 5:**
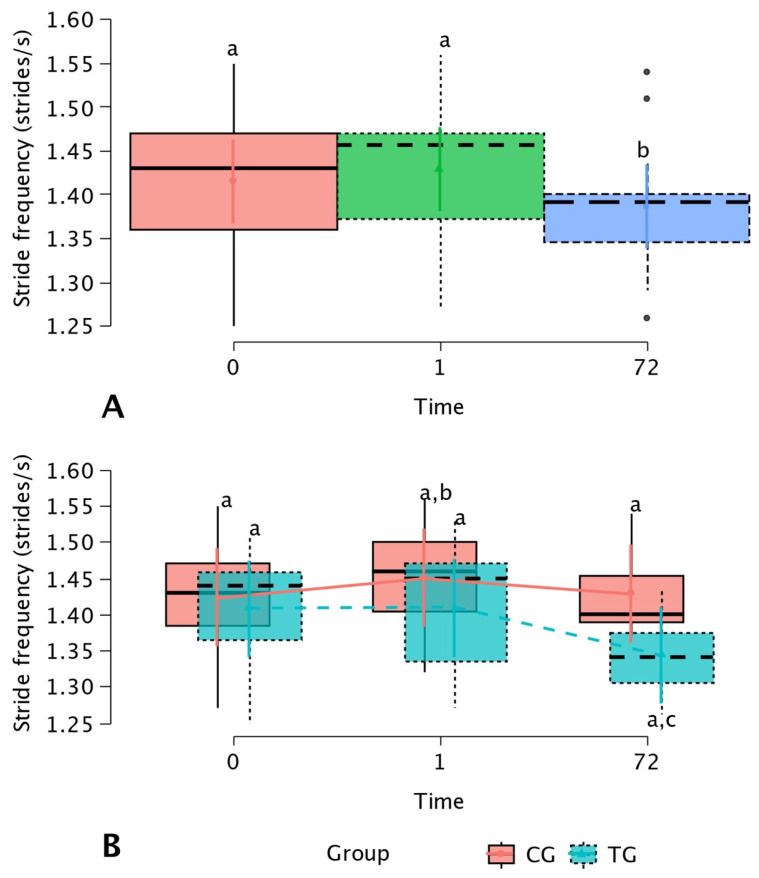
Box plots showing the mean and 95% mean confidence interval (95% CI) of stride frequency (SF) (strides/s) according to the time factor (**A**), and SF according to the interaction between the treatment and time-fixed factors (**B**). ^a–c^ Lowercase letters denote statistically (*p* < 0.05) significant differences between the different groups.

Regarding the effect of the time factor, the SF was significantly (*p* < 0.05) the lowest at 72 h when compared with before (T0) and after (T1) the DN treatment (Figure 5A). On the other hand, the SF registers of the TG at 72 h were significantly lower when compared with the values for the same variable of the CG after treatment (T1) (*p* < 0.05) (Figure 5B).

### 3.2. Asymmetry Push-Off Phase

The treatment and time-fixed factors and their interactions did not significantly influence the FHROM, HPROM, and HPROM severity variables, while the treatment factor significantly (*p* = 0.015) influenced the FHROM severity variable (Table 2).

**Figure 6:**
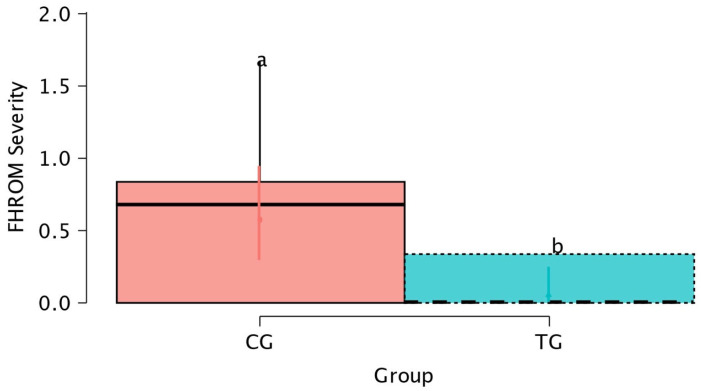
Box plots showing the mean and 95% CI for the forelimb head range of motion (FHROM) severity according to the treatment factor. ^a,b^ Lowercase letters denote statistically (*p* < 0.05) significant differences between the different groups.

Notably, the FHROM severity variable was significantly (*p* = 0.015) greater in the CG than in the TG (Figure 6).

### 3.3. Asymmetry Impact Phase

The FHROM, HPROM, HPROM severity, and FHROM severity variables were not significantly influenced by the treatment and time-fixed factors and their interactions (Table 3).

## 4. Discussion

To understand the impact of TrPs on biomechanical patterns, it is essential to first explore the underlying mechanisms involved in their formation. Simons’ integrated hypothesis stands as the most widely accepted model explaining the development of TrPs [37]. This theory suggests that muscle overuse or stress leads to ischemia and the release of pain-inducing substances. Consequently, this stimulates heightened acetylcholine activity, which leads to excessive muscle contraction, the formation of taut bands, and the activation of pain receptors, ultimately resulting in the development of TrPs.

Taut bands can disrupt the normal sequence of muscle activation in humans [38]. Signs of muscle damage include prolonged impairment or a reduction in muscle function, delayed onset of muscle soreness, stiffness, and swelling [39]. Additionally, the contracture related to a taut band also results in a reduction in the range of motion [8].

In dogs, the ongoing presence of TrPs is attributed to the same muscle-related factors that contribute to their development, primarily due to chronic muscle overload from mechanical stresses [8]. In horses, a chronic history of trauma or stress from heavy training can lead to MPS, often termed “overuse syndrome” [6]. It may be assumed that MPS is more common in sport horses. Nevertheless, the welfare of horses involved in equine-assisted therapies continues to be a focus of scientific interest [40]. The repetitive and extended work patterns of the assessed horses were believed to impact the development of TrPs and, as a result, their gait quality.

In humans, DN has been shown to be effective at reducing pain, increasing the range of motion, and improving muscle strength and coordination [41]. Its efficacy is explained through its physiological effects. In the taut band, the insertion of a needle at the endplate region may lead to a reduction in available acetylcholine stores, resulting in a lesser spontaneous electrical activity. The effects on blood flow involve the release of vasoactive substances, such as calcitonin gene-related peptide and substance P, which lead to vasodilation in small vessels and an increased blood flow. Regarding central sensitization, DN may stimulate both large myelinated fibers (i.e., Aβ- and Aδ-fibers) and C-fibers indirectly via the release of inflammatory mediators. As a result of mechanical stimulation, Aβ- and Aδ-fibers send afferent signals through the dorsolateral tracts of the spinal cord and may activate supraspinal and higher centers involved in pain processing [41]. However, no studies have yet evaluated these physiological effects in horses.

The two biomechanical parameters assessed by the app used in this study were the SF and limb asymmetry. The asymmetry was assessed by measuring the vertical displacement of the head and pelvis at a trot [1]. These vertical displacements are key indicators for the visual detection of lameness [33]. However, the clinical presentation of lameness is variable and some gait irregularities may not consistently or easily result in detectable lameness [42]. These issues are common in physical therapy and rehabilitation consultations. Due to the lack of standardized measures of gait quality in the field, a recent survey identified 24 parameters of in-hand gait that could serve as the basis for a simple, field-based outcome measure [43]. The challenge of using this app to detect gait patterns beyond traditional lameness and to demonstrate the effectiveness of DN through gait analysis was significant.

In the various models run in our study, we observed that the time factor significantly influenced the SF variable, but interestingly, the interaction between the treatment and time factors showed that the DN-treated horses had the lowest SF values at 72 h post-treatment compared with both the pre- and post-treatment measurements after the DN intervention. This finding might indicate that the SF parameter may be useful to evaluate this type of intervention in horses. It is important to consider that the SF has been investigated as an indicator of gait quality in dressage horses, with a slower SF being favored by judges [23,24]. In lame horses, some studies have reported an increase in SF and stance duration when the horse was forced to maintain a trot speed. These horses exhibited faster and shorter strides, which could be attributed to a reduction in the duration of the swing phase [44].

In the present study, non-significant differences were observed for both asymmetry push-off and impact phase variables, except for the FHROM severity, which was significantly affected by the treatment factor, which demonstrated that horses treated with DN had lower values of FHROM severity compared with the horses in the CG. During the intervention, the brachiocephalicus muscle showed a higher frequency and number of LTRs compared with the other needled muscles. This may explain a greater therapeutic effect on the neck and forelimb functionality and, consequently, a lower FHROM severity in the treated group compared with the control group. The quantification of LTRs was based on clinical observations made during the intervention. Therefore, the relationship between the number of LTRs and the DN could be explored in future studies as a potential predictor of the efficacy of the technique.

In this research, the effectiveness of the DN was only evaluated in the short term. In humans, a review presented by Fernández de las Peñas [19] indicated that the effects were primarily observed in the short term, with the effect sizes ranging from moderate to slight. Therefore, this review suggests integrating DN with pain neuroscience education, graded exercise, and manual therapy [19]. This leads to the consideration that a multimodal approach in horses might also be the most effective option for achieving optimal therapeutic outcomes.

The muscles evaluated and treated in this investigation are essential contributors to biomechanical function. The brachiocephalicus facilitates lateral neck flexion and forelimb protraction, while the trapezius contributes to scapular swinging movements [45]. The gluteus medius, biceps femoris, and semitendinosus are involved in hip extension, which is accompanied by an active stifle extension facilitated by concentric contraction of the quadriceps femoris [45]. In our clinical experience, TrPs are often identified in the muscles studied. This agrees with Ridgway [2,6], who indicated that the trapezius, brachiocephalicus, gluteal, and hamstring groups are the most common muscles to develop TrPs.

Manual palpation of taut bands to identify TrPs has been described in muscle assessment [9]. Although the evaluation of pain was beyond the scope of this study, it is a critical component in the detection of TrPs. Pain cannot be verbally communicated by animals [46], and referred pain is difficult to evaluate [9]. However, the presence of a taut band and behaviors indicative of pain during palpation have been documented in horses [12] and dogs [3,5]. Facial expressions are used as indicators of pain, including asymmetrical ears, tension around the eyes, a tense stare, strained nostrils and mouth, and tension in the mimic and chewing muscles [47]. These facial indicators were observed in this study to identify TrPs during manual palpation in the muscles evaluated.

There were some limitations to this study. The sample size was small, and larger studies involving a broader range of patients are necessary. Standardizing lighting conditions during recordings is essential. This study was conducted in an outdoor arena with adequate lighting, as recording in an indoor facility with controlled lighting was not feasible. However, the variation in the intensity of sunlight, at times, could affect the quality of the video recordings. Another potential limitation could be the model of the mobile device used. The application guidelines suggest that a three-camera system is optimal. A new iPhone 14, which met the technical requirements, was utilized in this study; however, it was equipped with two cameras. A Pro model with a three-camera system may have been more suitable.

This study had several strengths. The assessments were conducted under real clinical conditions, and a control group was included. A consistent and uniform sand surface that was familiar to the horses was used during all recordings. Additionally, the study population consisted of horses with recognized social and therapeutic value to humans.

Further research should explore larger sample sizes to confirm these preliminary findings and identify the long-term effects of DN. Moreover, future studies could investigate the potential of this tool for monitoring rehabilitation protocols and evaluating the efficacy of different therapeutic techniques. It would also be valuable to include conditions such as lunge work and rider interaction, as these better reflect real-world training and rehabilitation scenarios. 

## 5. Conclusions

This pilot clinical trial demonstrated the effectiveness of the DN technique in improving the gait quality in horses. The AI-based gait analysis app proved to be a valuable tool for evaluating this technique. Among the parameters evaluated, the SF was identified as the most appropriate to evaluate the DN technique. Further research is needed to integrate different types of work and techniques to better reflect the field of equine rehabilitation. 

## Figures and Tables

**Table 1:** Generalized linear mixed models (GLMM) results showing the effects of the treatment, and time-fixed factors and their interactions for the stride frequency variables evaluated in this study.

Variable	Factor (Effect)	df	ChiSq	*p*
Number of front strides	Treatment	1	1.501	0.221
	Time	2	0.302	0.860
	Treatment × time	2	2.695	0.260
Variation of front strides	Treatment	1	0.351	0.554
	Time	2	0.192	0.909
	Treatment × time	2	0.191	0.909
Number of hind strides	Treatment	1	3.277	0.070
	Time	2	3.196	0.202
	Treatment × time	2	2.086	0.352
Variation of hind strides	Treatment	1	0.103	0.748
	Time	2	0.211	0.900
	Treatment × time	2	0.214	0.899
Stride frequency	Treatment	1	1.389	0.239
	Time	2	12.261	0.002
	Treatment × time	2	8.383	0.015

**Table 2:** GLMM results showing the effects of the treatment and the time-fixed factors and their interactions for asymmetry push-off phase variables evaluated in this study.

Variable	Factor (Effect)	df	ChiSq	*p*
FHROM (%)	Treatment	1	0.117	0.732
	Time	2	1.209	0.546
	Treatment × time	2	4.898	0.086
FHROM severity	Treatment	1	6.540	0.011
	Time	2	5.467	0.065
	Treatment × time	2	5.451	0.066
HPROM (%)	Treatment	1	0.649	0.420
	Time	2	0.237	0.888
	Treatment × time	2	2.632	0.268
HPROM severity	Treatment	1	0.426	0.514
	Time	2	0.281	0.869
	Treatment × time	2	0.290	0.865

**Table 3:** GLMM results showing the effects of the treatment and the time-fixed factors and their interactions for the asymmetry impact phase variables evaluated in this study.

Variable	Factor (Effect)	df	ChiSq	*p*
FHROM (%)	Treatment	1	0.355	0.551
	Time	2	1.798	0.407
	Treatment × time	2	0.393	0.822
FHROM severity	Treatment	1	0.363	0.547
	Time	2	0.718	0.698
	Treatment × time	2	0.825	0.662
HPROM (%)	Treatment	1	0.341	0.559
	Time	2	3.886	0.143
	Treatment × time	2	0.062	0.969
HPROM severity	Treatment	1	0.111	0.739
	Time	2	0.497	0.780
	Treatment × time	2	0.185	0.912

## Data Availability

The raw data supporting the conclusions of this article will be made available by the authors upon request.

## Supplementary Materials

The following supporting information can be downloaded from https://www.mdpi.com/article/10.3390/ani15111517/s1, Table S1: Individual data of the horses included in this study.

## Abbreviations

The following abbreviations are used in this manuscript:

MPS	Myofascial pain syndrome
TrPs	Trigger points
DN	Dry needling
AI	Artificial intelligence
app	Smartphone application
TG	Treatment group
CG	Control group
SF	Stride frequency
FHROM	Forelimb head range of motion
LTRs	Local twitch responses
HPROM	Hindlimb pelvic range of motion
GLMMs	Generalized linear mixed models

## Appendix A

**Table A1:** Muscle anatomical references, actions, and needle size used for treatment.

Muscle	Anatomical References	Action	Needle Size (mm)
Brachiocephalicus	Origin: humeral crest. Insertion: mastoid process of the temporal bone.	Protraction of the forelimb. Bilateral: flexion of the neck. Unilateral: lateroflexion and rotation of the neck and poll.	0.30 × 40
Trapezius (cervical part)	Origin: nuchal and supraspinous ligament of C2–T10. Insertion: spine of the scapula.	Draws scapula cranially and upward.	0.25 × 25
Gluteus medius	Origin: longissimus dorsi, ilium, sacrum, and sacroiliac and sacrotuberous ligaments. Insertion: greater trochanter of the femur.	Extension, abduction, and internal rotation of the coxo-femoral joint.	0.30 × 40
Biceps femoris	Origin: spinous and transverse processes of Cd 3–5 and sacrotuberous ligament (vertebral part). Ischial tuberosity (pelvic part). Insertion: patella, intermediate and lateral patellar ligaments, tibial crest, crural fascia, and calcaneus.	Cranial part: extension of the coxo-femoral and stifle joint, as well as abduction. Caudal part: flexion of the knee, abduction, and extension of the tarsal joint.	0.30 × 40
Semitendinosus	Origin: last sacral vertebra, transverse processes of Cd 1–2, and sacrotuberous ligament (vertebral part). Ischial tuberosity (pelvic part). Insertion: cranial border of the tibia, crural fascia, and common calcanean tendon.	During weight-bearing: extension of the stifle and tarsal joint. During non-weight-bearing: flexion of the stifle joint and draws the limb caudally.	0.30 × 40
Quadriceps femoris (vastus lateralis)	Origin: lateral surface of the femur. Insertion: lateral part of the anterior surface and base of the patella.	Extension of the stifle joint.	0.30 × 40
