# Supplementary material for: Short-Term Impact of Dry Needling Treatment for Myofascial Pain on Equine Biomechanics Through Artificial Intelligence-Based Gait Analysis
Source: Animals (Basel). 2025 May 22;15(11):1517. doi: 10.3390/ani15111517 (PMC12153734; doi:10.3390/ani15111517)
Supplement: Supplementary file 1 [file animals-15-01517-s001.zip › animals-3626581-supplementary.pdf]

Table S1. Individual data of the horses included in the study.

Animal	Age (Years)	Gender (M/F)	Coat	Breed	Discipline
1	20	M	Black	Quarter Horse	Equine-assisted therapy
2	26	M	Gray	Crossbreed	Equine-assisted therapy
3	18	F	Bay	Pure Spanish Horse	Equine-assisted therapy
4	15	F	Gray	Arabian x Jaca Navarra	Leisure Equine-assisted therapy
5	15	F	Gray	Crossbreed	Leisure
6	29	M	Bay	Crossbreed	Equine-assisted therapy
7	24	M	Bay	Crossbreed	Equine-assisted therapy
8	4	F	Bay	Spanish Sport Horse x KWPN	Leisure
9	16	M	Bay	Chilean Criollo	Leisure
10	16	F	Bay	Chilean Criollo	Equine-assisted therapy
11	19	F	Bay	Chilean Criollo	Equine-assisted therapy
12	26	M	Bay	Chilean Criollo	Equine-assisted therapy
13	19	F	Appaloosa	Appaloosa	Leisure
14	18	M	Gray	Pure Spanish Horse	Equine-assisted therapy
